# Acute effects of transcranial direct current stimulation (tDCS) on peak torque and 5000 m running performance: a randomized controlled trial

**DOI:** 10.1038/s41598-023-36093-5

**Published:** 2023-06-08

**Authors:** Leila Fernanda dos Santos, Devisson dos Santos Silva, Micael Deivison de Jesus Alves, Erika Vitoria Moura Pereira, Hortência Reis do Nascimento, Matheus Santos de Sousa Fernandes, Aristela de Freitas Zanona, Beat Knechtle, Katja Weiss, Felipe J. Aidar, Raphael Fabricio de Souza

**Affiliations:** 1 Department of Physical Education, Federal University of Sergipe (UFS), São Cristóvão, Brazil; 2 Graduate Program in Physical Education, Federal University of Sergipe (UFS), São Cristóvão, Brazil; 3 Group of Studies and Research of Performance, Sport, Health and Paralympic Sports—GEPEPS, Federal University of Sergipe (UFS), São Cristovão, Brazil; 4 Department of Occupational Therapy, Federal University of Sergipe (UFS), Lagarto, Sergipe Brazil; 5 Graduate Program in Neuropsychiatry and Behavioral Sciences, Federal University of Pernambuco (UFPE), Recife, Brazil; 6 Institute of Primary Care, University of Zurich, 8091 Zurich, Switzerland; 7 Medbase St. Gallen Am Vadianplatz, Vadianstrasse 26, 9001 St. Gallen, Switzerland

**Keywords:** Biophysical methods, Neuroscience, Neurology

## Abstract

The benefits of transcranial direct current stimulation (tDCS) on brain function, cognitive response, and motor ability are well described in scientific literature. Nevertheless, the effects of tDCS on athletes’ performance remain unclear. To compare the acute effects of tDCS on the running performance of 5000 m (m) runners. Eighteen athletes were randomized into Anodal (n = 9) groups that received tDCS for 20 min and 2 mA, and Sham (n = 9), in the motor cortex region (M1). Running time in 5000 m, speed, perceived exertion (RPE), internal load and peak torque (Pt) were evaluated. The Shapiro–Wilk test followed by a paired Student’s t-test was used to compare Pt and total time to complete the run between the groups. The running time and speed of the Anodal group (p = 0.02; 95% CI 0.11–2.32; d = 1.24) was lower than the Sham group (p = 0.02, 95% CI 0.05–2.20; d = 1.15). However, no difference was found in Pt (p = 0.70; 95% CI − 0.75 to 1.11; d = 0.18), RPE (p = 0.23; 95% CI − 1.55 to 0.39; d = 0.60) and internal charge (p = 0.73; 95% CI − 0.77 to 1.09; d = 0.17). Our data indicate that tDCS can acutely optimize the time and speed of 5000 m runners. However, no alterations were found for Pt and RPE.

## Introduction

Transcranial direct current stimulation (tDCS) is a neuromodulation technique that delivers a constant, low-intensity flow of electric current to the scalp^1^. Additionally, it can promote increased cortical excitability after anodic stimulation^2^, resulting in changes in the resting membrane potential of the target neurons^3^. Benefits of tDCS on the brain and cognitive functions have already been identified^4^ and were also associated with improved motor skills^1,5,6^.

Stimuli in the motor cortex (M1) region can directly influence sport performance^7^. Evidence indicates that tDCS can promote psychophysiological and physical performance changes, enabling improvements in countermovement jump performance after 20 min (min) of stimulation^8^ and increased exhaustion time in cyclists’ performance on a 10 km (km) course after a 13 min of stimulation^7^. An improvement in the endurance performance of isolated muscle groups in isometric tests has also been reported^9^.

However, some studies failed to identify any acute stimulation effect on M1. In amateur runners they showed no improvement in performance with 15 min of 2 mA stimulation^10^ and the kicking ability of taekwondo athletes after 15 min of stimulation at the intensity of 2 mA^11^. Perceived Exertion (RPE) in recreational runners did not change in a treadmill test after 20 min of tDCS^12^ as well as in countermovement jumps in healthy subjects^13^. As well as in an evaluation performed in runners with a 30 m sprint and in RPE, with 15 min and 2 mA stimulation in the dorsolateral prefrontal cortex, no improvement in perceived exertion and performance was found^14^. We can then consider the stimulation time, the intensity applied, and the assembly of the electrodes as factors that differ in previous research, causing the results to diverge, instigating possibilities for obtaining positive results regarding the use of tDCS in running, since the studies have not yet been exhausted.

During running, the amplitude of individual muscle activation and groupings can vary. Incomplete muscle activation occurs consistently during exhaustive and high-resistance exercise^15^. The identification of performance responses of runners related to the application of tDCS remains inconclusive. We believe that tDCS applied acutely can potentiate the muscular activation of the lower limbs, which can be identified with the peak torque. As well as minimize the perception of effort.

The present study’s main objective was to explore an anodic electrode’s effects on the M1 and the performance and RPE in 5000 m runners. The secondary objective was to investigate the effect of tDCS on the knee extensor muscles’ peak torque (Pt) that are fundamental for runners. Therefore, we hypothesized that tDCS at an intensity of 2 mA would improve running times after a 20 min application.

## Materials and methods

### Participants

Twenty-four runners participating in the running club of the Universidade Federal de Sergipe (UFS) were eligible for the study. The study was designed to be single-blinded, randomized and counter-balanced. Randomization was done using Microsoft Excel 2021 software. Blinding was achieved by telling all participants that they would receive stimulation. We included runners older than 18 years, with a weekly frequency of three to five workouts, at an average pace of 5000 m ≤ 4:30 min/km^16^, without any neuromuscular, skeletal, or cardiovascular contraindication, and with a minimum of 12 months of running experience.

**Figure 1:**
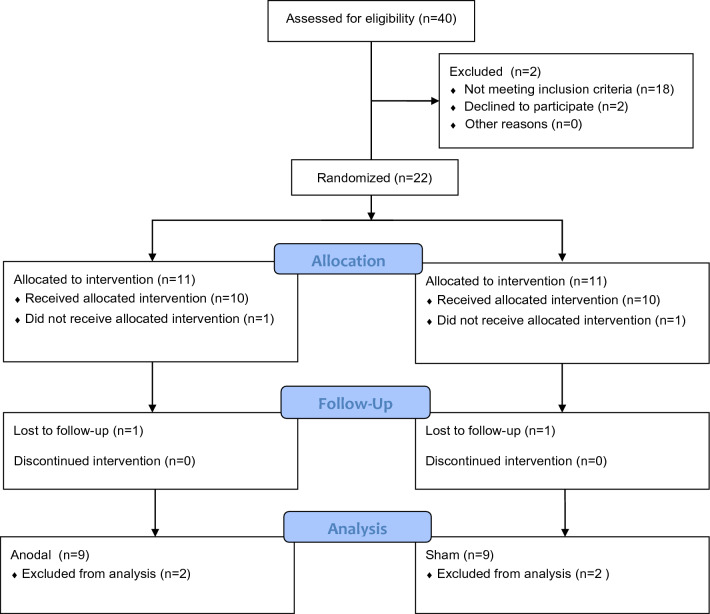
Description of sample selection and randomization^17^.

We excluded subjects who (a) did not finish the course due to upper respiratory tract complications, (b) reported muscle pain during warm-up and if they reported fear of electrostimulation (in the case of two runners). The runners were randomized into two groups: Anodal (n = 9; 29 ± 7 years; 63 ± 8 kg) and Sham (n = 9; 25 ± 4 years; 66 ± 12 kg). All runners were blinded. The entire flow chart of the research can be seen in (Fig. 1).

### Ethical consideration

The entire procedure was explained to the athletes, who signed the informed consent form. The study was approved by the Ethics Committee on Human Beings of UFS (CAAE: 56703722.2.0000.5546), according to the Helsinki declaration, with Brazilian Clinical rials Registry, (03/27/2023), (RBR-4yt3pvc).

### Experimental protocol

**Figure 2:**
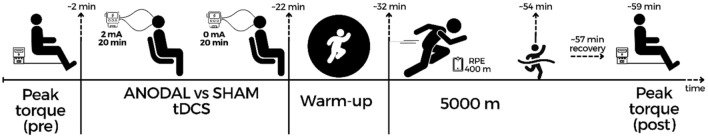
Experimental design—*tDCS* transcranial direct current stimulation. Peak torque is performed, then brain stimulation, followed by warm-up of the run, a slight recovery back to peak torque.

The athletes visited the Physical Education Department—athletics track at UFS to perform a familiarization session on the procedure and data collection. Initially, we collected the peak torque in three trials, with 30 s of recovery between them. The tDCS was performed for 20 min at an intensity of 2 mA. After a 10-min warm-up, the runners started a 5000-m race on an official athletic track. At the end of the run, there was a 3-min rest, after which the peak torque was reevaluated. The survey design can be seen in (Fig. 2).

### Transcranial direct current stimulation (tDCS)

The tDCS was applied according to the guidelines proposed by Vitor-Costa et al.^7^ and Angius et al.^1^. A Microestim Genius electrostimulator, manufacturer NKL, ANVISA registration 80191680008, dimensions 13.7 cm × 8.2 cm × 4.2 cm, was used. The electrodes were placed on the M1 (soaked in saline solution), with the anode at points C3 and C4 located according to the 10–20 stimulation system and the cathode on the occipital protuberance, with a current intensity of 2 mA for 20 min. The electrical current was modulated for 30 s, and the stimulator display monitored the electrical resistance. For the Sham group, the procedure and electrode setup were the same. However, the electrostimulator was turned off one minute after the start.

### Peak torque

Peak torque (Pt) was evaluated as the maximum isometric torque generated by the knee extensor muscles. The Pt was determined by multiplying the peak isometric force and the length of the segment, given by the distance between the attachment point of the load cell cable and the center of the knee joint. For this evaluation, a load cell (Kratos model CZC500) fixed on an inextensible cord and attached near the malleolus by means of a Velcro system positioned next to the malleoli was used^18^.

### Running test

After the stimulation, the athletes were instructed to start the 5000 m course on the official athletics track at the command of the sound signal. The time to complete the course was recorded using a manual stopwatch (Vollo® VL-512) as well as the average speed of each athlete was observed, through the total time to complete the race.

### Perceived effort and internal load of the training session

The CR-10^19^ perceived exertion scale was used to assess perceived exertion (RPE) at each 400 m lap. To calculate the session internal charge, Foster’s proposal^20^ was used, which is the value of the perceived effort obtained after the stress test and warm-up (total session time in minutes).

### Statistics

The normality of the data was assessed by the Shapiro–Wilk test followed by a paired Student’s t-test to compare the Pt and the total time to complete the course between the groups. The RPE and internal charge at each lap were examined by two-way analysis of variance (ANOVA). Tukey’s post-hoc test for multiple comparisons was used whenever necessary. The Cohen d test was used to assess the effect size, adopting the cutoff points of 0.02–0.15 for a small effect, 0.16 to 0.35 as a medium effect, and greater than 0.35 as a large effect. Spearman’s correlation verified the link between RPE and tDCS. We performed a statistical power analysis a priori to estimate the appropriate number of participants required to generate these results. Using G Power program (3.1.9.7), we calculated an effect size (f = 1.5) with 95% confidence (power = 0.95) and α err prob (0.05) in ANOVA repeated measures and between factors. A 95% confidence interval was adopted with a significance value of p < 0.05. The statistical data were tabulated using JAMOVI v. 2.3 software.

### Ethics approval and consent to participate

The study was approved by the Ethics Committee on Human Beings of Universidade Federal de Sergipe (CAAE: 56703722.2.0000.5546), according to the Helsinki declaration.

## Results

### Sample characterization

Table 1 shows the sample characterization, in which the Anodal group athletes were 28 ± 7 years old, 174 ± 1 cm high, with a body mass of 62 ± 8 kg and a BMI of 21 ± 2 kg/m^2^. The Sham group athletes were 29 ± 10 years old, 171 ± 1 cm high, with a body mass of 65 ± 10 kg and a BMI of 22 ± 2 kg/m^2^.

**Table 1:** Characterization of the sample.

Participants ANODAL	Age (years)	Body mass (kg)	Height (cm)	BMI (kg/m^2^)	Participants *SHAM*	Age (years)	Body mass (kg)	Height (cm)	BMI (kg/m^2^)
A1	36	69	173	23	A0	26	65	169	23
B1	29	70	189	20	B0	20	63	169	22
C1	24	59	170	20	C0	25	58	177	19
D1	42	66	174	22	D0	31	66	170	23
E1	25	73	178	23	E0	24	51	166	19
F1	25	59	167	21	F0	34	65	169	23
G1	40	71	170	25	G0	23	89	185	26
H1	24	59	178	19	H0	26	68	175	22
I1	21	45	166	16	I0	26	64	174	21
Mean	29	63	173	21		25	67	173	22
SD	7	8	6	2		4	12	8	2

*BMI* body mass index.

### Primary outcomes

**Figure 3:**
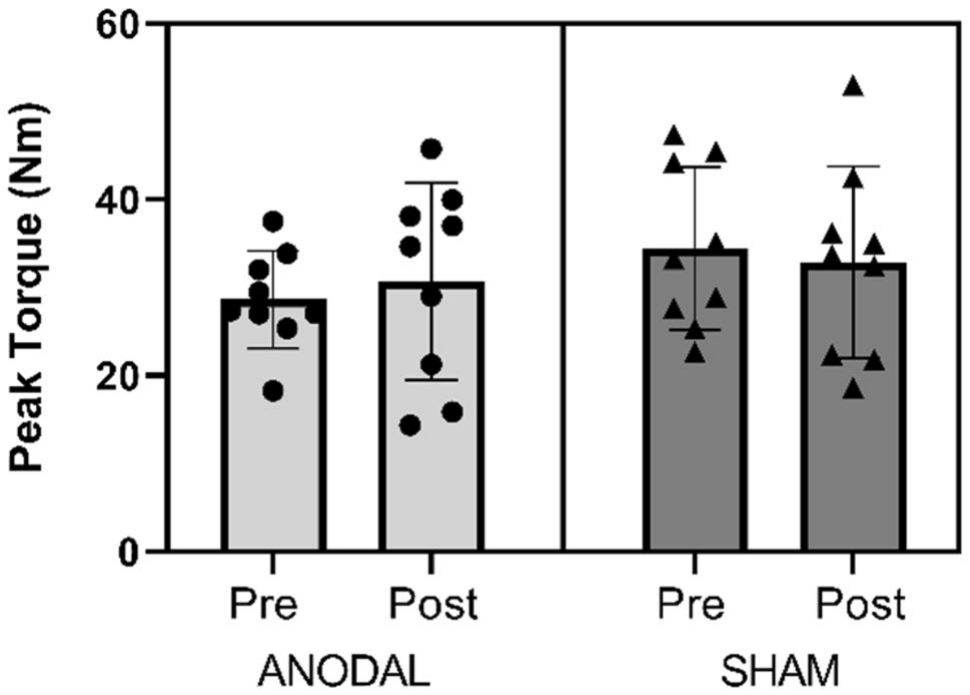
Peak torque (Nm) pre and post 5000 m of running and tDCS with 2 mA intensity; ANODAL (n = 9) and SHAM (n = 9). All values are presented as mean ± standard deviation. p = 0.70.

**Figure 4:**
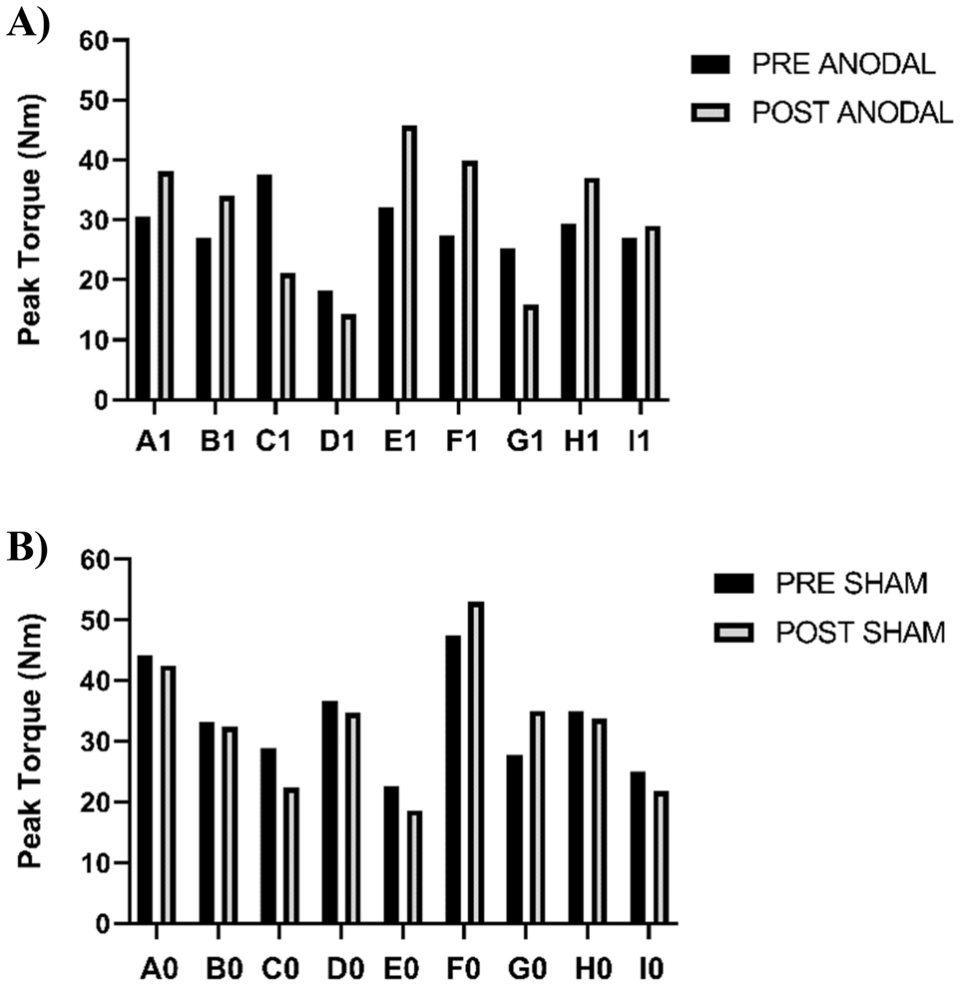
Peak torque (Nm) pre and post 5000 m of running and tDCS with 2 mA intensity, the individual values; ANODAL (n = 9) and SHAM (n = 9).

No difference was observed between the groups for peak torque (p = 0.70; 95% CI ± 1.11; d = 0.18). Figure 3 presents the pre- and post-values of both groups. Figure 4 shows the individual values of the runners at peak torque, showing minimal variation between the pre and post results.

**Figure 5:**
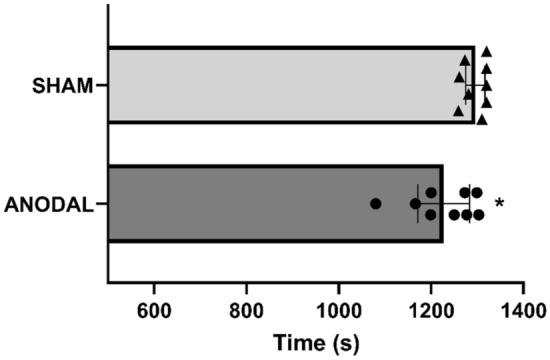
Total time of the 5000 m run. ANODAL (n = 9) and SHAM (n = 9). Values are presented as mean ± standard deviation. *p = 0.02.

**Figure 6:**
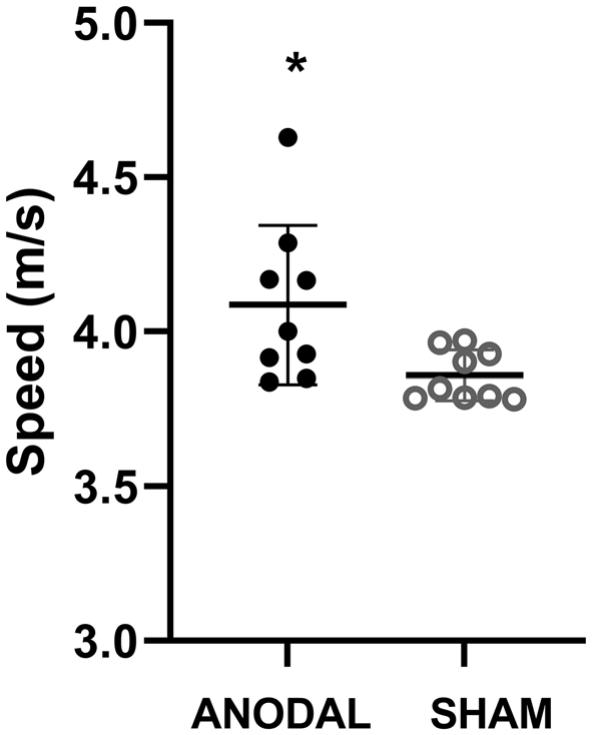
Average speed achieved. ANODAL (n = 9) and SHAM (n = 9). Values are presented as mean ± standard deviation. *p = 0.02.

The Anodal group showed a decrease in running time (p = 0.02; 95% CI 0.11–2.32; d = 1.24) on the 5000 m course compared to the Sham group. The absolute difference identified was 69 s. Figure 5 shows the mean and individual running times. The average speed is presented in Fig. 6 (= 0.02, 95% CI 0.05–2.20; d = 1.15), in which the runners in the Anodal group performed significantly higher than the Sham group.

### Secondary outcomes

**Figure 7:**
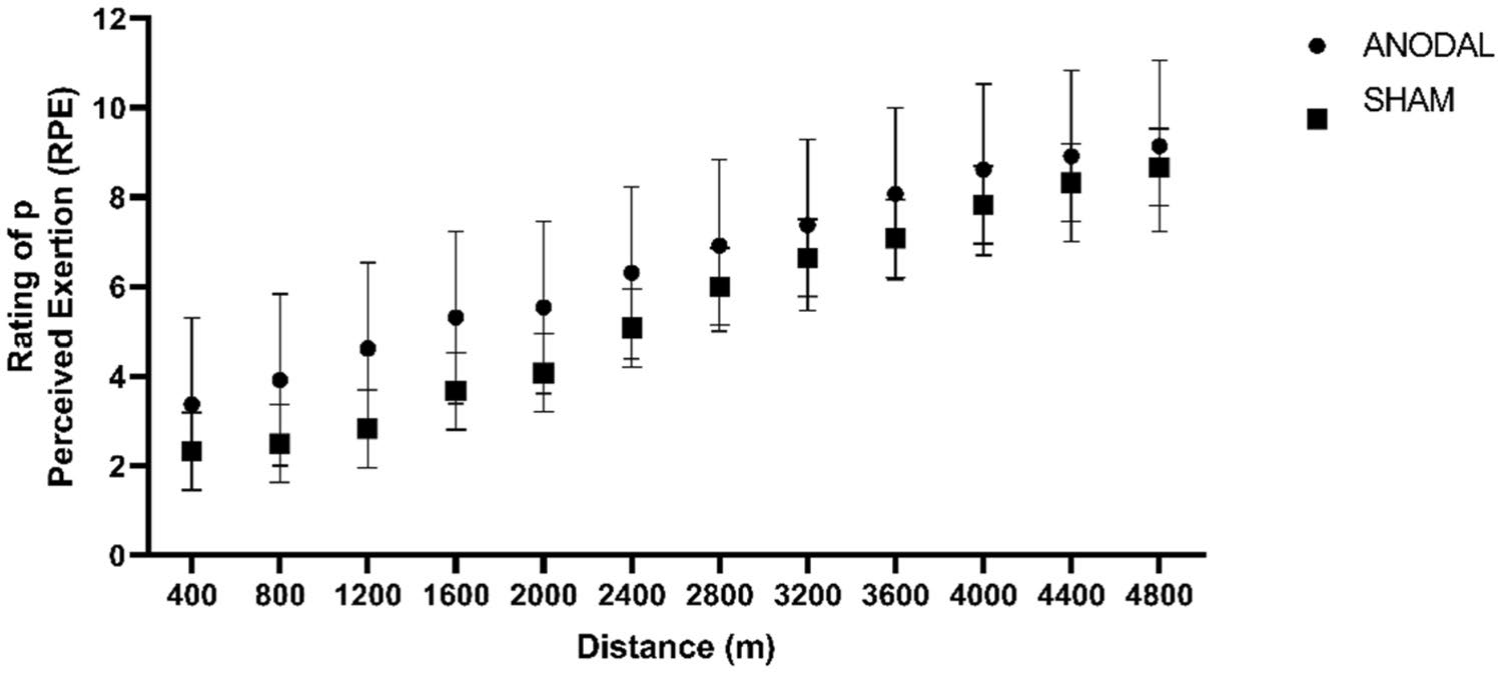
Perception of Effort at each 400 m lap. Values are presented as mean ± standard deviation. ANODAL (n = 9) and SHAM (n = 9). p = 0.23.

**Figure 8:**
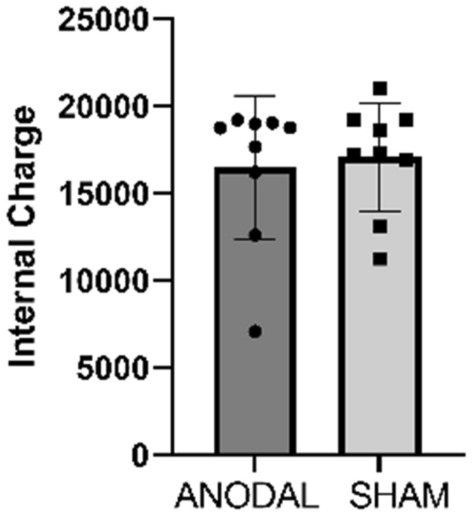
Internal Load. ANODAL (n = 9) and SHAM (n = 9). Values are presented as mean ± standard deviation. p = 0.73.

There was no difference in RPE between the groups (F [1,11] = 1.60, p = 0.23 95% CI − 1.55 to 0.39; d = 0.60). Similarly, the internal load showed no difference (F [1,14] = 0.12, p = 0.73; 95% CI − 0.77 to 1.09; d = 0.17). Figures 7 and 8 illustrates the values of PE and internal load respectively.

## Discussion

The objective of the present study was to analyze the effects of tDCS on 5000 m running performance, muscle strength and RPE in runners. The results show that 20 min of stimulation with an intensity of 2 mA on the primary motor cortex promoted improved athletic performance by reducing the running time in the Anodal group, with no changes observed for RPE, internal load, and peak torque.

The mechanisms of action related to the modulation of neuronal activity induced by tDCS are not yet fully understood^21^. However, possible explanations for the reduction in running time are related to increased neuronal excitability sufficient to modify neuronal membrane potentials. This can promote changes in the firing rate of neurons by increasing motor stimuli^22^. Other studies also indicate that tDCS may influence synaptic functionality by changing the activity of neurotransmitters and their receptors, triggering different neuronal plasticity processes, including long-term potentiation (LTP)^23^.

Long-term effects of tDCS may be associated with changes in protein synthesis and gene expression^24^. In addition, changes in the blood flow after stimulation were verified in neuroimaging studies in the study of Zheng et al. These findings were potentially related to tDCS, evidencing an increased oxygen supply in cortical areas and subsequent increased neuronal excitability^25^.

In our research, we found that 20 min of pacing can show improvement in running time. However, the results of similar studies still do not show a literature consensus. Recreational runners were found to have no improvement in fatigue after 15 min of stimulation with 2 mA at M1^10^. Runners being evaluated in a high-intensity, short-duration activity looking for acute effects on sprinting and on perceived exertion were not found effective results for performance enhancement with 15 min of stimulation with 2 mA^26^, the electrodes were mounted in the left dorsolateral prefrontal cortex. Similarly, taekwondo athletes did not improve kick potential after the same tDCS time in M1^11^. On the other hand, an increase in exercise tolerance in cycling athletes was seen with 13 min stimulations with 2 mA at M1^7^. More studies are needed to better understand the duration of stimulation and its effects.

The use of tDCS on the motor cortex has been related to motor development and fatigue tolerance^27^. Furthermore, evidence indicates that tDCS can improve the ability of the nervous system to produce muscle strength during maximal efforts^28^. In contrast, our results did not demonstrate any significant difference in peak torque. Corroborating other studies that did not obtain changes in the isometric strength pattern of the leg extension movement after anodic stimulation^10,29^. Thus, different tDCS protocols could be tested since an improvement in leg extensor muscle torque was found in two stimulation sessions seven days apart^30^.

Another factor that may be related to the lack of improvement in peak torque in our study was the maximum effort performance during the course. It is known that the ability of muscles to generate force becomes progressively impaired during maximum effort and a gradual recovery occurs at the end of physical effort^26^. Furthermore, the degree of strength reduction and recovery duration depends on the intensity and type of exercise performed^31^. In our study, the runners performed the peak torque assessment at the beginning and shortly after the end of the 5000 m.

It is known that RPE and internal load analysis are subjective. Our expectation was to find a significant improvement in the athletes’ RPE as well, considering that there was an improvement in their running performance. The function responsible for the regulation and control of attention is reached by performing an exercise that causes effort and pain^32^, however, it was not possible to evidence a reduction in the RPE. Therefore, we believe that the stimulation of the motor cortex has little influence on subjective decisions, such as the RPE, because they are related to the regions of the cerebral cortex responsible for feelings and emotions^33^. As there is also a huge inter-individual variability, such as genetics, cranial and cerebral anatomy, psychological state, organization of inhibitory and excitatory circuits, and neurotransmitter activity^34^.

We observed self-consciousness as an important point in deciding the stimulation strategy during exercise; in this sense, RPE in each exercise can help the individual better understand the momentary tension caused by physical exertion helping the individual decide to modulate exercise intensity^35^.

There are limitations to our study to be considered. We believe that the evaluation of peak torque could have been performed on days other than the 5000 m run, furthermore it could be evaluated immediately after tDCS. If performed on the same day, we suggest longer recovery intervals to gradually return muscle strength after maximal effort. Sample size per group, the lack of crossover design and lack of a third group could be better recommended. We also believe that an investigation with recreational athletes may still be opportune since they present different sensitivities after stimulation because of the lower excitability threshold than trained runners. Further research should be conducted to investigate the chronic effects of tDCS, thus deepening the knowledge regarding the amplitude of responses in performance in these protocols.

Finally, the improvements in physical performance related to the acute protocol investigated here can be of great importance for the day-to-day training of athletes. This result implies practical considerations, mainly due to the possibility of access to stimulation and its cost benefit.

## Conclusion

We conclude that the use of tDCS in an acute form improved the performance of 5000 m runners but showed no significant influence on RPE and peak torque.

## Data Availability

The datasets used and/or analysed during the current study are available from the corresponding author on reasonable request.
