# Author Correction: Acute effects of transcranial direct current stimulation (tDCS) on peak torque and 5000 m running performance: a randomized controlled trial

**DOI:** 10.1038/s41598-023-42345-1

**Published:** 2023-10-02

**Authors:** Leila Fernanda dos Santos, Devisson dos Santos Silva, Micael Deivison de Jesus Alves, Erika Vitoria Moura Pereira, Hortência Reis do Nascimento, Matheus Santos de Sousa Fernandes, Aristela de Freitas Zanona, Beat Knechtle, Katja Weiss, Felipe J. Aidar, Raphael Fabricio de Souza

**Affiliations:** 1 Department of Physical Education, Federal University of Sergipe (UFS), São Cristóvão, Brazil; 2 Graduate Program in Physical Education, Federal University of Sergipe (UFS), São Cristóvão, Brazil; 3 Group of Studies and Research of Performance, Sport, Health and Paralympic Sports—GEPEPS, Federal University of Sergipe (UFS), São Cristovão, Brazil; 4 Department of Occupational Therapy, Federal University of Sergipe (UFS), Lagarto, Sergipe Brazil; 5 Graduate Program in Neuropsychiatry and Behavioral Sciences, Federal University of Pernambuco (UFPE), Recife, Brazil; 6 Institute of Primary Care, University of Zurich, 8091 Zurich, Switzerland; 7 Medbase St. Gallen Am Vadianplatz, Vadianstrasse 26, 9001 St. Gallen, Switzerland

Correction to: *Scientific Reports* 10.1038/s41598-023-36093-5, published online 08 June 2023

The original version of this Article contained an error. The way the individual values were represented in Figure 6 did not represent the individual results correctly. The original Figure 6 and the accompanying legend appear below.

**Figure 6:**
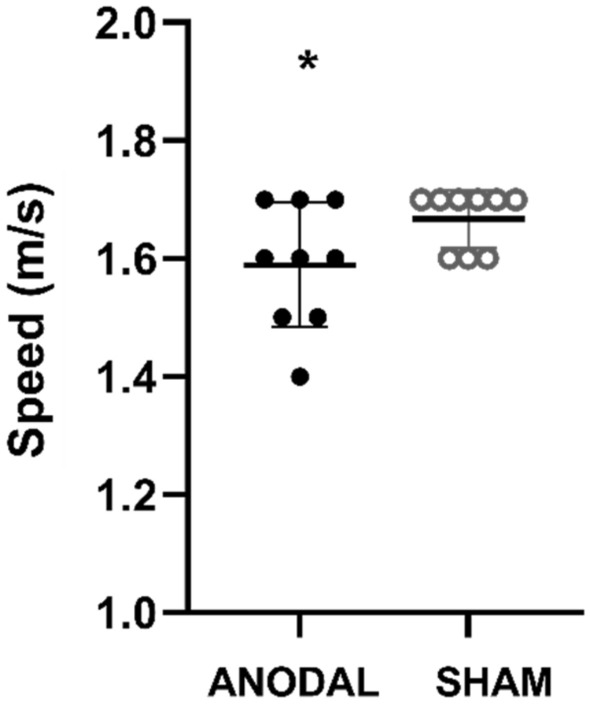
Average speed achieved. ANODAL (n = 9) and SHAM (n = 9). Values are presented as mean ± standard deviation. *p = 0.02.

The original Article has been corrected.

